# Predictors of HBsAg seroclearance in HBeAg-negative chronic hepatitis B patients treated with nucleotide analogs plus polyethylene glycol interferon

**DOI:** 10.3389/fmed.2024.1510230

**Published:** 2025-01-08

**Authors:** Yan Peng, Mingzhe Ma, Ting Liu, Wenmin He, Shutao Lin, Wa Zhong, Xiaohui Min

**Affiliations:** ^1^Department of Gastroenterology, Sun Yat-sen Memorial Hospital, Sun Yat-sen University, Guangzhou, China; ^2^Department of Infectious Disease, Sun Yat-sen Memorial Hospital, Sun Yat-sen University, Guangzhou, China; ^3^Department of Gastroenterology, Shenshan Medical Center, Memorial Hospital of Sun Yat-sen University, Shanwei, China; ^4^Department of Infectious Diseases, Shenshan Medical Center, Memorial Hospital of Sun Yat-sen University, Shanwei, China

**Keywords:** nucleoside analogs, pegylated interferon, HBeAg-negative, chronic hepatitis B, HBsAg clearance

## Abstract

**Introduction:**

The minority of the chronic hepatitis B (CHB) patients received polyethylene glycol interferon (PEG-IFN) combined with nucleotide analogs (NAs) can obtain hepatitis B surface antigen (HBsAg) clearance.

**Methods:**

In order to find out the advantaged population, we retrospectively collected 122 CHB patients treated with NAs alone or NAs plus PEG-IFN for 48 weeks, who were admitted to Sun Yat-sen Memorial Hospital from 2019 to 2024.

**Results:**

We found HBsAg clearance rate in NAs plus PEG-IFN group was 40.98%, which was significantly higher than that in the NAs group. Thus, NAs plus PEG-IFN therapy served as a relatively ideal regimen and the patients received combined treatment were then incorporated for further analysis for searching efficacy predictors. Through using univariate and multivariate analysis, we confirmed the predictive value of HBsAg, alanine aminotransferase (ALT) at week 24, and ALT change values from baseline to week 24. The area under the receiver operating characteristic (ROC) curve of each indicators ranged from 0.663 to 0.982.

**Discussion:**

In conclusion, our study verified the clinical value of NAs plus PEG-IFN for treating CHB patients. Moreover, for the first time, we found ALT change values from baseline to week 24 (dALT2) could act as a novel independent clinical efficacy predictors in the forementioned population.

## 1 Introduction

Chronic hepatitis B (CHB) is a serious global public health problem. In 2019, it was estimated that 296 million people in the world are hepatitis B surface antigen (HBsAg) positive, and the global infection rate was about 3.5% (1). CHB viral infection is an important cause of cirrhosis and hepatocellular carcinoma, with approximately 30% of cirrhosis cases and 45% of hepatocellular carcinoma cases in the world caused by chronic hepatitis B infection. Previous studies have shown that HBsAg clearance can reduce the prevalence of cirrhosis, liver cancer and all-cause mortality in CHB patients (2, 3). The current ideal therapeutic endpoint for CHB is clinical cure, also known as functional or immunologic cure, which is defined as the completion of a limited course of therapy with persistent undetectable serum HBsAg and Hepatitis B virus (HBV) DNA, negative hepatitis B e antigen (HBeAg), with or without HBsAg seroconversion, persistence of residual covalently closed circular DNA (cccDNA), remission of liver inflammation and improvement in liver histopathology and a significant reduction in the incidence of end-stage liver disease (4–6).

Antiviral therapy is the mainstay of prevention of CHB progression, and antiviral agents currently used for CHB treatment include two major classes: nucleoside analogs (NAs) and polyethylene glycol interferon (PEG-IFN). There are six main NAs approved for CHB treatment: entecavir, tenofovir alafenamide fumarate, tenofovir disoproxil fumarate, adefovir, telbivudine, lamivudine (7). NAs inhibit viral replication by inhibiting the activity of HBV DNA polymerase, with low drug resistance and few adverse effects, and have been the first choice of treatment for CHB patients for many years. However, it is difficult to obtain HBsAg clearance through NAs monotherapy, and some studies have found that the HBsAg clearance rate obtained by NAs monotherapy is less than 1% (8). The direct antiviral effect and special immunomodulatory effect of PEG-IFN make it play an important role in HBsAg clearance, and many clinical studies have confirmed the efficacy of interferon in CHB patients, especially in the dominant population of interferon therapy, and HBsAg clearance can be achieved with interferon treatment alone (9). Recently, new strategies of combing NAs with PEG-IFN have dramatically improved the achievement of loss/seroconversion in CHB patients. However, on the one hand, most of the patients remained HBsAg positive at the end of combination therapy; on the other hand, they suffered from side effects of PEG-IFN (10). Thus, finding out the advantaged population who can benefit from NAs plus PEG-IFN therapy has become a hot research topic. In this study, we retrospectively analyzed the efficacy of NAs combined with PEG-IFN in treating CHB patients and its related influencing factors.

## 2 Materials and methods

### 2.1 Inclusion and exclusion criteria

A total of 122 CHB patients attending Sun Yat-sen Memorial Hospital from 2019 to 2024 were collected and divided into NAs group (*n* = 61) and NAs plus PEG-IFN group (*n* = 61) according to the choice of treatment regimen. Subsequently, the NAs plus PEG-IFN group was divided into clear (*n* = 25) and non-clear (*n* = 36) groups depending on whether HBsAg was cleared.

Inclusion criteria:

Chronic HBV infection with negative HBeAg according to diagnostic criteria (7). That was to say, HBsAg and/or HBV DNA were positive for more than 6 months and HBeAg was negative.
(2) 18–60 years old, HBV DNA ≤ 500 IU/mL, HBsAg < 1,500 IU/mL.
Those who had no contraindication to interferon therapy and were willing to accept PEG-IFN therapy signed the informed consent form.

Exclusion criteria:

1. Allergy to interferon.
2. Pregnant and lying-in women or those who plan to be pregnant within 2 years.
3. Combined with other viral liver diseases (such as HCV), autoimmune hepatitis (liver disease), alcoholic liver disease, fatty liver disease (metabolic related fatty liver), hepatolenticular degeneration.
4. Cirrhotic decompensation, or those who have ever had cirrhotic decompensation.
5. Liver cancer and other malignant tumors.
6. Patients with autoimmune diseases, psychosis and thyroid dysfunction (hyperthyroidism or hypothyroidism).
7. HIV co-infection.
8. Those who are using immunosuppressants.
9. Peripheral white blood cell count < 3.5 × 10^9^/L and/or platelet count < 80 × 10^9^/L.
10. Patients with serious important organs (such as cardiovascular, lung, kidney, brain lesions and fundus) lesions.

### 2.2 Treatment

Taking oral antiviral drugs (such as entecavir, tenofovir alafenamide fumarate, tenofovir disoproxil fumarate, lamivudine and telbivudine) with or without subcutaneous injection of PEG-IFNα-2b (Xiamen Tebao Bioengineering Pharmaceutical Co. State Pharmaceutical License No. S20160001, specification: 180 μg/ampoule, weekly) for 48 weeks.

### 2.3 Monitoring indicators

Quantification of HBV-DNA, HBsAg, HBsAb and Other serum biochemical indicators. These serum biomarkers included alanine aminotransferase (ALT), total bilirubin (TB), creatinine (Cr), phosphocreatine kinase (CK), fasting blood-glucose (FBG), phosphorus (P), leucocyte, neutrophil, erythrocyte, platelet, hemoglobin and thyroid function. These factors were recorded before treatment, meanwhile, some of them were recorded at 12, 24, 36, and 48 weeks after treatment.

### 2.4 Test method

HBV-DNA was detected by fluorescence quantitative PCR (internal standard quantitative method), and the lower limit of detection was 20 IU/mL. HBV markers (HBsAg, HBsAb, and HBeAg) were quantitatively detected by chemiluminescence. The detection limit of HBsAg was 0.00 IU/mL. HBsAg ≤ 0.05 IU/mL was negative, HBsAb ≥ 10mIU/mL was positive.

### 2.5 Statistical analysis

SPSS 25.0 was used for statistical analysis of the data, the count data were described by rate and percentage, and *χ*^2^ test was used for comparison between groups. The measurement data were tested for normal distribution, and the measurement data in line with normal distribution were described by *X* ± *S* (mean ± standard deviation). Two independent samples *t*-test was used for comparison between the two groups, and paired samples *t*-test was used for comparison before and after treatment. One-way analysis of variance (ANOVA) was used to compare multiple groups. Measurement data that did not conform to normal distribution were described by M (P25–P75) (median, upper and lower quartiles). Mann–Whitney *U* test was used for comparison between two groups, and Kruskal–Wallis *H* rank sum test was used for comparison between multiple groups. Univariate and multivariate logistic analysis were used to analyze the independent predictors of HBsAg clearance, and ROC curves were drawn to assess the predictive value. *p* < 0.05 (both sides) was considered statistically significant.

## 3 Results

### 3.1 Baseline data of the NAs and NAs plus PEG-IFN groups

A total of 122 patients were included in this study, 61 in the NAs group and 61 in the NAs plus PEG-IFN group (combination group). In the NAs group, all patients were taking only one NAs, with 38 using entecavir and 23 using tenofovir alafenamide fumarate. The demographic data and baseline indices of the two groups were shown below in Table 1. The male to female ratio was similar in both groups (*p* = 0.262) and there was no statistical difference in the age distribution (*p* = 0.462). HBV DNA level in both groups was less than 500 IU/mL and there were no statistically significant differences in the remaining baseline indicators as well.

**Table 1:** Baseline data of 122 patients with CHB.

Baseline characteristics (*N* = 122)	NAs (*N* = 61)	Combination (*N* = 61)	*P*
Sex			
Male	41	35	0.262
Female	20	26	
Age (year)	42 ± 8	43 ± 9	0.462
HBsAg (IU/ml)	218.70 (71.94–465.76)	80.71 (9.29–527.27)	0.077
ALT (U/L)	22 (16–30)	22 (18–35)	0.442
AFP (ng/ml)	2.24 (1.53–2.95)	2.41 (1.85–3.09)	0.154
TB (μmol/L)	14.2 (10.5–16.3)	12.3 (9.3–14.7)	0.064
P (mmol/L)	1.10 (0.86–1.32)	1.06 (1.0–1.15)	0.617
Cr (μmol/L)	85 ± 13	84 ± 18	0.716
CK (U/L)	142 (81–282)	133 (97–254)	0.802
FBG (mmol/L)	5.4 (5.0–5.7)	5.3 (5.0–5.7)	0.487

CHB, chronic hepatitis B; NAs, nucleotide analogs; HBsAg, hepatitis B surface antigen; ALT, alanine aminotransferase; AFP, alpha fetoprotein; TB, total bilirubin; P, phosphorus; Cr, creatinine; CK, creatine kinase; FGB, fasting blood sugar.

### 3.2 Comparison between the NAs and combination groups

One patient in the NAs group achieved HBsAg clearance, and 25 in the combination group, with a statistically significant difference in HBsAg clearance rates between the two groups (*p* < 0.001) (Table 2).

**Table 2:** Comparison of HBsAg clearance rate between the NAs and combination groups.

	NAs	Combination	*P*
HBsAg seroclearance	1	25	<0.001
HBsAg non-seroclearance	60	36	

NAs, nucleotide analogs; HBsAg, hepatitis B surface antigen.

According to the guidelines (7), the upper limits of normal (ULN) for ALT values are 30 U/L and 19 U/L for men and women, respectively. We examined the rate of ALT abnormality rate in the NAs group and the combination group at week0, 12 and 24. There was no statistically significant difference between the two groups at baseline, but there was a significant difference at week 12 and 24 (Table 3).

**Table 3:** Comparison of ALT abnormality rate between NAs group and combination groups.

Time	NAs	Combination	*P*
	(*N* = 61)	(*N* = 61)	
Week 0	8	9	0.794
Week 12	7	52	<0.001
Week 24	7	47	<0.001

NAs, nucleotide analogs.

As the ALT abnormality rate at week 12 and 24 was different between the NAs group and the combination group, we further quantified ALT at different time points. The baseline ALT levels were similar between two groups, but there were statistically significant differences in the ALT levels at week 12 and 24 (Table 4). Moreover, the combination group had higher ALT levels at week 12 and 24, whereas there were no significant changes in ALT levels at week 0, 12, and 24 in the NAs group (Tables 5, 6).

**Table 4:** Comparison of ALT between the NAs group and the combination group at weeks 0, 12, 24.

Time	NAs	Combination	*P*
	(*N* = 61)	(*N* = 61)	
Week 0	22 (16–30)	22 (18–35)	0.442
Week 12	21 (16–30)	72 (48–91)	<0.001
Week 24	19 (15–30)	72 (41–103)	<0.001

NAs, nucleotide analogs.

**Table 5:** Comparison of ALT between week 0 and week 12 in the NAs and combination groups.

Group	ALT0w	ALT12w	*P*
NAs	22 (16–30)	21 (16–30)	0.796
Combination	22 (18–35)	72 (48–91)	<0.001

NAs, nucleotide analogs; ALT, alanine aminotransferase.

**Table 6:** Comparison of ALT between week 0 and week 24 in the NAs and combination groups.

Group	ALT0w	ALT24w	*P*
NAs	22 (16–30)	19 (15–30)	0.208
Combination	22 (18–35)	72 (41–103)	<0.001

NAs, nucleotide analogs; ALT, alanine aminotransferase.

### 3.3 Comparison of baseline data between the HBsAg seroclearance group and the HBsAg non-seroclearance group

Since only one patient in the NAs group achieved HBsAg clearance, combination with PEG-IFN therapy obviously served as a priority selection for CHB patients. Thus, we incorporate the combination group for further analysis to explore the factors influencing the achievement of HBsAg clearance. Patients received NAs plus PEG-IFN therapy were categorized into the HBsAg seroclearance group and the HBsAg non-seroclearance group according to whether they achieved HBsAg clearance or not. There were 25 patients in the HBsAg seroclearance group (male/female: 13/12) and 36 patients in the HBsAg non-seroclearance group (male/female: 22/14), and there was no significant difference in gender ratio between the two groups (*p* = 0. 344). The mean age of the HBsAg seroclearance group was 41 ± 8 years, and the mean age of the HBsAg non-seroclearance group was 44 ± 9 years. The age distribution between the two groups was also not significantly different (*p* = 0.165). Analysis of the baseline serological indices of the two groups revealed statistically significant differences in HBsAg and FGB levels between the two groups (*p* = 0.032 and *p* = 0.017, respectively), as shown in Table 7.

**Table 7:** Comparison of baseline data between the HBsAg seroclearance and HBsAg non-seroclearance groups.

Baseline characteristics	HBsAg seroclearance group	HBsAg non-seroclearance group	*P*
(*N* = 61)	(*N* = 25)	(*N* = 36)	
Gender			
Male	13	22	0.479
Female	12	14	
Age (year)	41 ± 8	44 ± 9	0.165
HBsAg (IU/ml)	57.01 (2.91–204.49)	236.61 (11.46–699.14)	0.032
DNA (IU/ml)	20 (20–37.5)	35 (20–100)	0.218
ALT (U/L)	21 (17–35)	23 (19–35)	0.607
AFP (ng/ml)	2.43 (1.925–3.140)	2.400 (1.818–3.018)	0.538
TB (μmol/L)	12.5 (8.7–14.0)	12.0 (9.4–16.10)	0.719
P (mmol/L)	1.09 (1.005–1.150)	1.05 (1.00–1.14)	0.480
Cr (μmol/L)	84 ± 20	83 ± 16	0.789
CK (U/L)	133 (106–268)	136 (97–188)	0.449
FGB (mmol/L)	5.1 (4.9–5.4)	5.5 (5.1–5.7)	0.017

HBsAg, hepatitis B surface antigen; DNA, deoxyribonucleic acid; ALT, alanine aminotransferase; AFP, alpha fetoprotein; TB, total bilirubin; P, phosphorus; Cr, creatinine; CK, creatine kinase; FGB, fasting blood sugar.

### 3.4 Analysis of HBsAg clearance rate and clearing time

At the end of the follow-up, 25 of the 61 CHB patients achieved HBsAg clearance (male/female: 13/12). The overall HBsAg clearance rate was 40.98% (25/61), 37.14% (13/35) for males, and 46.15% (12/26) for females, with no statistically significant difference in the HBsAg clearance rate of males and females (*p* = 0.479). The median age of the population was 43 years old, and the subjects were divided into ≤40 years old group and >40 years old group according to their age. The HBsAg clearance rate of the two groups were 44% (11/25) and 38.89% (14/36) respectively, and there was no significant difference between the two groups (*p* = 0.690). In the HBsAg HBsAg seroclearance group, the median weeks of HBsAg conversion and the median weeks of HBsAg clearance were both 36 weeks. The median weeks of HBsAg conversion were 36 weeks for men and 18 weeks for women and the median weeks of HBsAg clearance were 48 weeks and 24 weeks for men and women, respectively. In comparison of women and men, the median weeks of HBsAg conversion and HBsAg clearance were both statistically different (*p* = 0.019 and *p* = 0.021, respectively).

### 3.5 Comparison of HBsAg clearance rate at different baseline HBsAg levels

**Figure 1:**
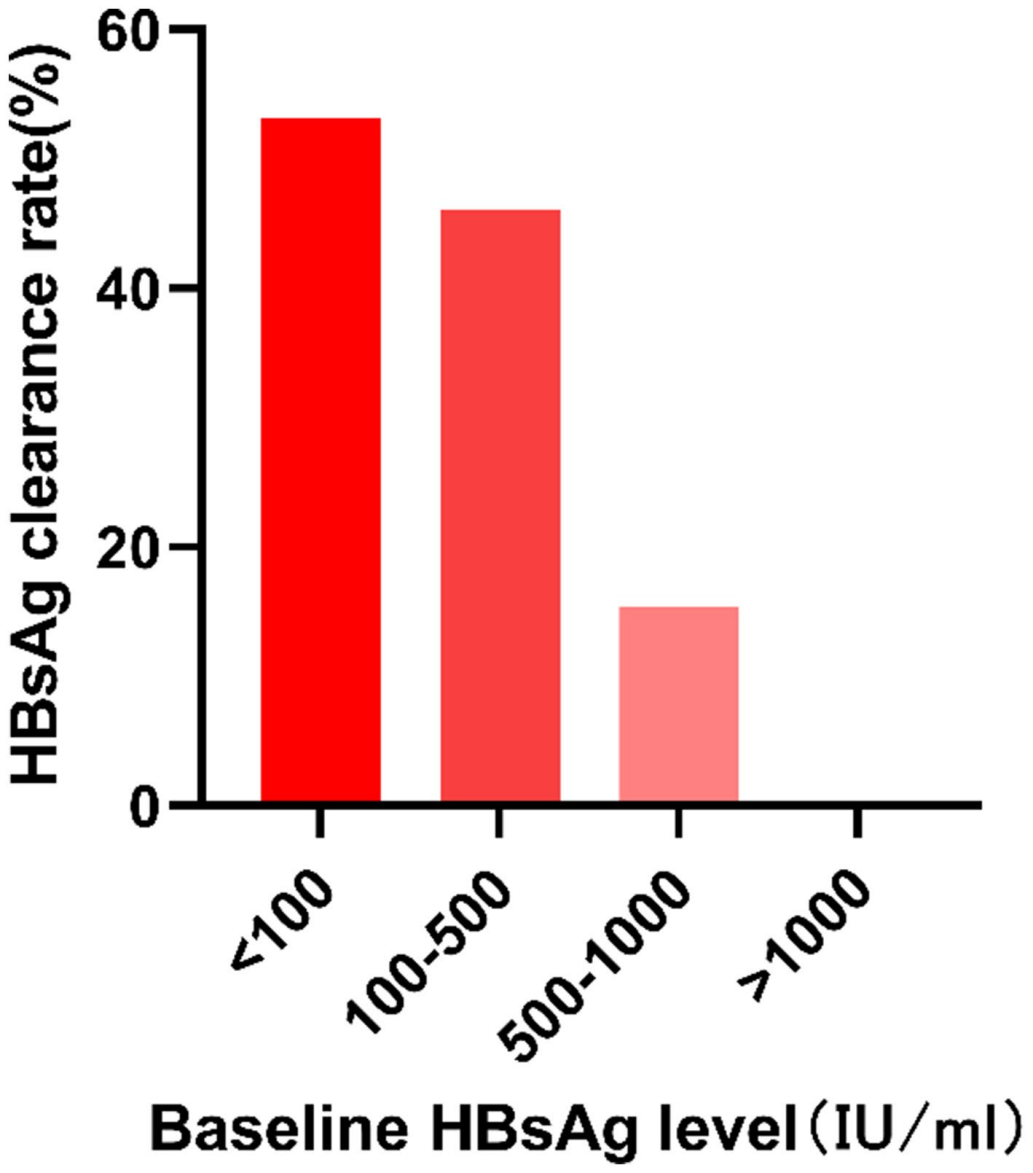
Comparison of HBsAg clearance rate in different baseline HBsAg levels group. HBsAg, hepatitis B surface antigen.

With reference to previous similar studies, the patients received NAs plus PEG-IFN therapy were divided into four groups according to their baseline HBsAg level: HBsAg < 100 IU/mL group, 100 ≤ HBsAg < 500 IU/mL group, 500 ≤ HBsAg < 1,000 IU/mL group and HBsAg ≥ 1,000 IU/mL group (as shown in Figure 1; Table 8). There were 32, 13, 13, and 3 patients in the four groups respectively, and 17, 6, 2, and 0 patients in each group, respectively, achieved HBsAg clearance, with a HBsAg clearance rate of 53.13, 46.15, 15.38, 0%, there was a significant difference in the HBsAg clearance rate among the four groups (*p* = 0.047). Further pairwise comparison showed that HBsAg < 100 IU/mL group was statistically different from 500 ≤ HBsAg < 1,000 and HBsAg ≥ 1,000 IU/mL group (*P2* = 0.02 and *P3* = 0.039, respectively). No statistically significant differences were observed in the comparisons between the rest of the groups. There was also a difference in the HBsAg clearance rate between HBsAg < 100 IU/mL group and HBsAg ≥ 100 IU/mL group (*p* = 0.043).

**Table 8:** Comparison of HBsAg clearance rate in different baseline HBsAg level groups.

HBsAg (IU/ml)	HBsAg seroclearance group (*N* = 25)	HBsAg non-seroclearance group (*N* = 36)	HBsAg clearance rate	*P*
HBsAg < 100	17	15	53.13%	0.047
100 ≤ HBsAg < 500	6	7	46.15%	
500 ≤ HBsAg < 1,000	2	11	15.38%	
1,000 ≤ HBsAg	0	3	0%	

P1 = 0.672 (HBsAg < 100 vs. 100 ≤ HBsAg < 500).

P2 = 0.02 (HBsAg < 100 vs. 500 ≤ HBsAg < 1,000).

P3 = 0.039 (HBsAg < 100 vs. 1,000 ≤ HBsAg).

P4 = 0.202 (100 ≤ HBsAg < 500 vs. 500 ≤ HBsAg < 1,000).

P5 = 0.408 (100 ≤ HBsAg < 500 vs. 1,000 ≤ HBsAg).

P6 = 0.344 (500 ≤ HBsAg < 1,000 vs. 1,000 ≤ HBsAg).

HBsAg, hepatitis B surface antigen.

### 3.6 Comparison of changes in ALT between HBsAg seroclearance group and HBsAg non-seroclearance group

In order to study the changes of ALT in the course of NAs plus PEG-IFN therapy, the levels of ALT at baseline, week 12 and week 24 of HBsAg seroclearance group and HBsAg non-seroclearance group were studied in depth. As shown in Table 9, there was no statistically significant difference in the baseline ALT levels between the HBsAg seroclearance group and HBsAg non-seroclearance group. At week 12 and week 24, there was a significant difference in ALT between the two groups. The change of ALT level during treatment was further analyzed, as shown in Table 10. Three change values were calculated: dALT1 = ALT12w-ALT0w dALT2 = ALT24w-ALT0w dALT3 = ALT24w-ALT12w. The three change values of the ALT were compared between the two groups, and the three change values of ALT were different.

**Table 9:** Comparison of ALT at week 0, 12, and 24.

Time	HBsAg seroclearance group	HBsAg non-seroclearance group	*P*
	(*N* = 25)	(*N* = 36)	
Week 0	21 (17–35)	23 (19–35)	0.607
Week 12	92 (81–109)	51 (36–71)	*<0.001*
Week 24	112 (98–137)	47 (32–66)	*<0.001*

HBsAg, hepatitis B surface antigen; ALT, alanine aminotransferase. *P* < 0.001 means there is a significant difference in ALT between the HBsAg seroclearance group and HBsAg non-seroclearance group at week 12 and week 24.

**Table 10:** Comparison of changes in ALT.

dALT	HBsAg seroclearance group	HBsAg non-seroclearance group	*P*
	(*N* = 25)	(*N* = 36)	
dALT1	52 (30–74)	26 (9–43)	*0.003*
dALT2	71 (37–82)	19 (6–40)	*<0.001*
dALT3	18 (−19–40)	-4 (−20–13)	*0.028*

dALT1 = ALT12w-ALT0w; dALT2 = ALT24w-ALT0w; dALT3 = ALT24w-ALT12w.

HBsAg, hepatitis B surface antigen; ALT, alanine aminotransferase. *P* = 0.003, *P* < 0.001 and *P* = 0.028 mean the three change values of the ALT are different in the HBsAg seroclearance group and HBsAg non-seroclearance group.

### 3.7 Predictors of HBsAg clearance

#### 3.7.1 Univariate logistic regression analysis

In order to analyze the predictive factors affecting HBsAg clearance, the HBsAg levels at week 0, 12, 24, and 36, the levels of ALT at week 12 and 24, the change values of ALT 1, 2, 3, gender and age were included in the univariate logistic regression analysis (Table 11). Gender (*p* = 0.480) and age (*p* = 0.167) had no statistical significance in predicting HBsAg clearance. HBsAg at 12w (OR = 0.980, 95% CI = 0.999–1.001, *p* = 0.980) was also not statistically different. The remaining indicators were statistically different.

**Table 11:** Univariate logistic regression analysis.

Factors	OR	95% CI		*P*
		Lower	Upper	
Gender	1.451	0.517	4.070	0.480
Age	0.957	0.898	1.019	0.167
HBsAg0w	0.998	0.996	1.000	0.021
HBsAg12w	0.980	0.999	1.001	0.980
HBsAg24w	0.964	0.935	0.995	0.023
HBsAg36w	0.417	0.204	0.850	0.016
ALT12w	1.093	1.043	1.146	<0.001
ALT24w	1.179	1.057	1.316	0.003
dALT1	1.061	1.030	1.093	<0.001
dALT2	1.085	1.042	1.130	<0.001
dALT3	1.037	1.013	1.061	0.002

dALT1 = ALT12w-ALT0w; dALT2 = ALT24w-ALT0w; dALT3 = ALT24w-ALT12w.

HBsAg, hepatitis B surface antigen; ALT, alanine aminotransferase; OR, odds ratio, CI, confidence interval.

#### 3.7.2 Multivariate logistic regression analysis

The nine statistically significant indicators (HBsAg0w, HBsAg24w, HBsAg36w, ALT12w, ALT24w, dALT1, dALT2, and dALT3) obtained from the above univariate analysis were further included in the multivariate logistic regression analysis, in which HBsAg0w (OR = 0.998, 95% CI = 0.996–1.000, *p* = 0.021), HBsAg24w (OR = 0.964, 95% CI = 0.933–0.997, *p* = 0.017), ALT24w (OR = 1.179, 95% CI = 1.057–1.316, *p* = 0.003), and dALT2 (OR = 1.085, 95% CI = 1.042–1.130, *p* < 0.001) were statistically significant indicators, as shown in Table 12.

**Table 12:** Multivariate logistic regression.

Factors	OR	95% CI		*P*
		Lower	Upper	
HBsAg0w	0.998	0.996	1.000	0.021
HBsAg24w	0.964	0.933	0.997	0.017
ALT24w	1.179	1.057	1.316	0.003
dALT2	1.085	1.042	1.130	<0.001

HBsAg, hepatitis B surface antigen; ALT, alanine aminotransferase; OR, odds ratio; CI, confidence interval.

#### 3.7.3 ROC curve

**Figure 2:**
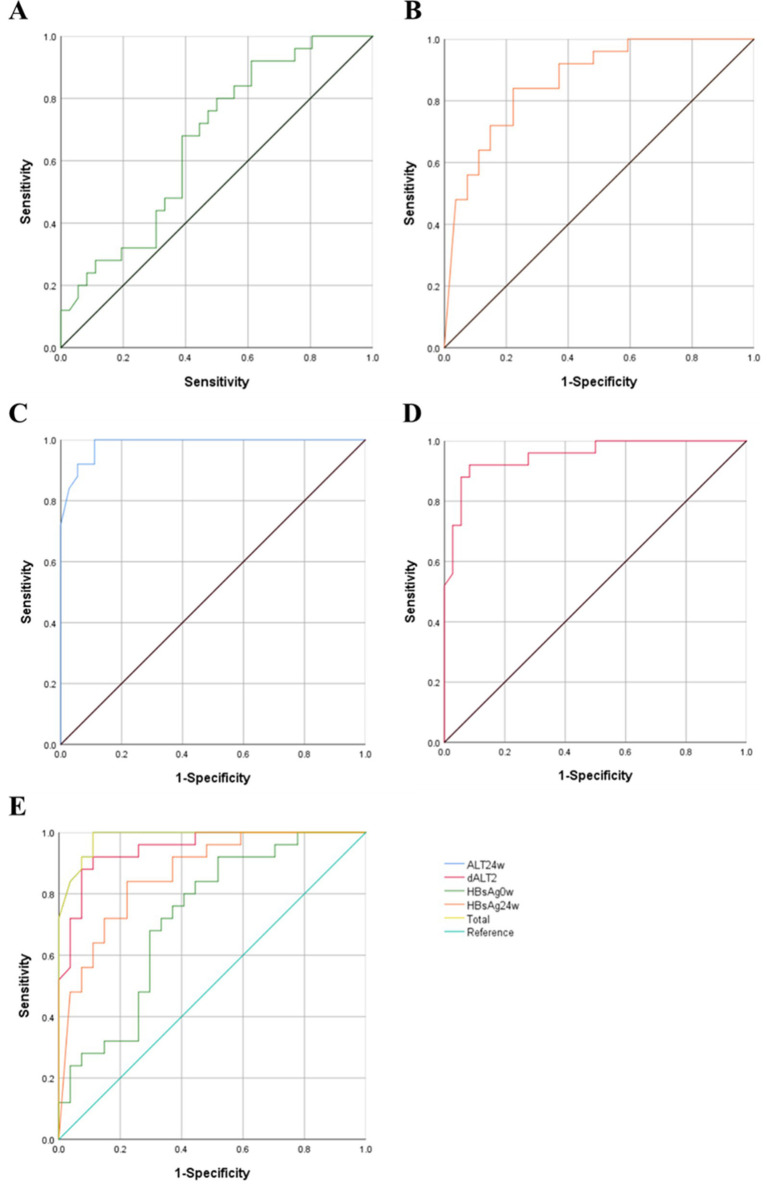
ROC curves of each predictors. **(A)** HBsAg0w-ROC Curve. **(B)** HBsAg0w-ROC Curve. **(C)** ALT24w-ROC Curve. **(D)** dALT2-ROC Curve. **(E)** Summary of ROC curves. ROC, receiver operating characteristic; HBsAg, hepatitis B surface antigen; ALT, alanine aminotransferase; OR, odds ratio; CI, confidence interval.

Multivariate logistic regression analysis showed that HBsAg0w, HBsAg24w, ALT 24w, dALT2 were independent predictors of HBsAg clearance. In order to furtherly analyze the predictive value of each factor, ROC curves were drawn as shown in Figures 2A–E. The area under the ROC curve of HBsAg 0w was 0.663 (95% CI = 0.584–0.862, *p* = 0.006). The area under the ROC curve of HBsAg 24w was 0.865 (95% CI = 0.768–0.962, *p* < 0.001). The area under the curve was 0.982 (95% CI = 0.957–0.998, *p* < 0.001) for ALT24w and 0.949 (95% CI = 0.894–0.996) for dALT2. Apparently, ALT24w has the largest area under the curve and the greatest predictive value, the area under the ROC curve of ALT24w and dALT2 were all greater than 0.9, which mean that all of them had good predictive value, the area under the combined prediction curve was 0.986 (95% CI = 0.959–0.999, *p* < 0.001), the sensitivity was 0.968, and the specificity was 0.887 (Table 13).

**Table 13:** ROC curve related statistical values of each predictor.

Test variables	AUC	*P*	95% CI		Sensitivity	Specificity
			Lower	Upper		
HBsAg0w	0.663	0.006	0.584	0.862	0.920	0.389
HBsAg24w	0.865	<0.001	0.768	0.962	0.840	0.778
ALT24w	0.982	<0.001	0.957	0.998	0.964	0.889
dALT2	0.949	<0.001	0.894	0.996	0.920	0.917
Total	0.986	<0.001	0.959	0.999	0.968	0.887

ROC, receiver operating characteristic; AUC, area under curve; HBsAg, hepatitis B surface antigen; ALT, alanine aminotransferase; OR, odds ratio; CI, confidence interval.

## 4 Discussion

Interferon has been initially used in the treatment of CHB since 1977 and was formally approved by the U.S. Food and Drug Administration (FDA) in 1986, and its alpha-type interferon has become the standard drug for CHB treatment. Currently, the main interferons used in CHB treatment are conventional interferon-alpha and PEG-IFN. Polyethylene glycol (PEG) is an inactive hydrophilic polymer, which is covalently incorporated into active protein molecules through biopharmaceutical technology to increase molecular weight and prolong the metabolic half-life, which ensures that the drug maintains a more stable concentration in the bloodstream to avoid fluctuations in drug efficacy (11). In addition, PEGylation also reduces the direct contact between interferon and immune cells, lowering the immunogenicity and thus reducing the occurrence of adverse reactions. Interferon therapy generally does not cause serious and fatal adverse reactions. The most common adverse reactions caused by interferon are flu-like symptoms (such as fever, headache, muscle aches, malaise) (7). They can be alleviated by prophylactic use of NSAIDs and discontinuation of interferon. The next most common adverse effects, like bone marrow suppression and thyroid abnormality, can also be prevented and treated with the use of granulocyte colony-stimulating factor and thyroid hormone medications. In summary, PEG-IFN has more stable efficacy, longer dosing intervals, and fewer adverse reactions than conventional interferon, and thus has become the main recommended drug in the clinical treatment of CHB.

Interferon can induce hepatocytes to be in an antiviral state by regulating gene expression and protein translation, exerting non-cytolytic antiviral effects at several stages of the hepatitis B virus life cycle, inducing the recruitment of three cellular proteins that bind to the cccDNA microchromosome, altering the post-translational modifications of cccDNA-associated histones, and inhibiting cccDNA transcription that further inhibits serum HBsAg synthesis (12). Interferon-*α* may also inhibit serum HBsAg synthesis by inducing APOBEC3A (13) and ISG20 (14) to induce cccDNA degradation, and in addition to the individual cellular context, cell-to-cell spread of viral resistance is a mechanism to amplify interferon-induced antiviral responses, with interferon-α inducing the transfer of resistance to hepatitis B virus from hepatic non-parenchymal cells to hepatocytes via exosomes (15, 16). In addition to its direct anti-hepatitis B virus effect, interferon can also regulate the levels of interleukin 1, interleukin 2, and tumor necrosis factor through the cytokine network, promote the proliferation of cytotoxic T lymphocytes, and activate the immune activity of immune-active cells, such as NK cells, K cells, and macrophages, so that hepatitis virus-infected hepatocytes can be killed and the virus can be cleared (17).

Previous studies have shown that patients who achieve HBsAg clearance have a significantly lower risk of developing cirrhosis and liver cancer (9, 18–20). The 1-year HBsAg clearance rate of NAs treatment alone fluctuated between 0 and 3% (21). The majority of studies have shown that NAs combined with pegylated interferon can significantly improve the clearance rate of HBsAg in the treatment of chronic hepatitis B and is higher than NAs or interferon alone (22, 23). A total of 122 patients with chronic hepatitis B were enrolled in our study, including 61 in the NAs group and 61 in the PEG-IFN group. One person in the NAs group achieved HBsAg clearance, while 25 in the PEG-IFN group did so, and the HBsAg clearance rate at 48 weeks in the PEG-IFN group reached 40.98%, which was significantly higher than that in the NAs group. The HBsAg clearance rate of the study was higher than that of OSST (24) and NEW SWITCH (25) studies at 48 weeks (22.2–26.5%), which may be related to the fact that all of our enrolled patients were HBeAg-negative and had an initial HBV DNA level of less than 500 IU/mL.

The Interferon guideline (26) proposes baseline guided therapy (BGT) and response guided therapy (RGT), suggesting that the baseline indicators and the changes during treatment affect the treatment effect. We categorized patients into four groups according to baseline HBsAg levels in the PEG-IFN group: HBsAg < 100 IU/mL group, 100 ≤ HBsAg < 500 IU/mL group, 500 ≤ HBsAg < 1,000 IU/mL group and 1,000 IU/mL ≤ HBsAg group. The HBsAg clearance rates of each group was 53.13, 46.15, 15.38, and 0%, respectively, with statistical difference (*p* = 0.047). Taking HBsAg = 100 IU/mL as the cut-off point, the HBsAg clearance rate in the group with HBsAg < 100 IU/mL was significantly higher than that in the group with HBsAg ≥ 100 IU/mL (*p* = 0.043), which was comparable with the Broquetas et al. (27) and Mimura et al. (28) study: patients with HBeAg-negative and baseline HBsAg < 100 IU/mL have the highest chance of HBsAg disappearance. This indicates that the lower the baseline HBsAg level is, the higher the HBsAg clearance rate is. There may be two reasons: one is that the sustained low level of viral antigen may reduce the stimulation of viral antigen which is favorable to the recovery of host immune function (29). Another is the function of HBV-specific T cells is negatively correlated with serum HBsAg level and viral load (30). The lower the level of serum HBsAg, the higher the function of HBV-specific T cells, and the stronger the ability to clear HBV.

If CHB patients are treated with interferon-*α* indiscriminately, the clearance rate of HBsAg is low after 48 weeks of treatment (3.0% ~ 7.0%) (31, 32). Previous studies have shown low baseline HBV DNA levels (<2 × 10^6–8^ IU/mL), high ALT levels (≥2ULN) were associated with the effectiveness of interferon-alpha therapy. At the same time, other factors such as the degree of inflammation and fibrosis in the liver tissue, the genotype of HBV, and the sex and age of the patient may also have some influence on the efficacy of treatment (33, 34).

The baseline ALT levels was similar in the NAs group and the interferon group, but there were differences in the changes of ALT during treatment, with the interferon group having higher ALT levels at week 12 and 24, while there were no significant changes in ALT levels at week 0, 12, and 24 in the NAs group. This was related to the different mechanisms of action of NAs and interferon, where nucleoside analogs mainly block viral replication by inhibiting the activity of HBV DNA polymerase and do not destroy hepatocytes. Correspondingly, interferon activates the immune activity of immunoreactive cells such as NK cells, K cells and macrophages through direct antiviral and immunomodulatory effects, thus killing hepatitis virus-infected hepatocytes, and then causing a large amount of aminotransferase release (17). In order to explore the related factors that affect PEG-IFN therapy to achieve HBsAg clearance, univariate and multivariate logistic regression analysis were conducted. Univariate logistic regression analysis showed that HBsAg0w, HBsAg24w, HBsAg36w, ALT12w, ALT24w, dALT1, dALT2, and dALT3 were statistically significant. Further multifactorial logistic regression analysis revealed that HBsAg0w, HBsAg24w, ALT24w, and dALT2 were the independent predictors of HBsAg clearance, among which the area under the curve of ALT24w was the largest (AUC = 0.982), with the greatest predictive value, and the area under the curve of the four combined predictors amounted to 0.986. An observational study in China (35) found that age ≤ 33 years old, lower baseline, 12-week, and 24-week HBsAg levels, and ALT elevation ≥2 × ULN at 12 weeks of treatment were significant predictors of HBsAg clearance at 72 weeks, which was similar to the results of this study. Due to the fact that the patients included in the study were older, and the younger patients were fewer in number, our results could not reflect the effect of age on the efficacy of the treatment. Acute elevation of ALT is generally affected by host factors and viral factors. On the one hand, it is caused by immune mediation and is related to the decrease of HBV DNA level, leading to the seroconversion of HBeAg and HBsAg. On the other hand, it is related to progressive liver injury. Acute elevation of ALT during PEG-IFN treatment generally predicts a better therapeutic outcome (36). In this study, the elevation of ALT was the first scenario. The levels of ALT in the HBsAg seroclearance group were higher than those in the HBsAg non-seroclearance group at week 12 and 24 and all returned to normal after treatment. The number of viral genotype testing and liver biopsies performed in this study was small and not analyzed. Although changes in HBsAg levels and ALT have good predictive value for efficacy evaluation, emphasis should be placed on evaluating patients’ suitability for interferon therapy based on the combination of several indexes before initiating treatment, as well as evaluating the desirability of continuing interferon therapy based on the response to efficacy.

## 5 Conclusion

In conclusion, combination of PEG-IFN is expected to improve the HBsAg clearance rate of NAs-treated patients with low viral load (HBsAg < 100 IU/mL) 48 weeks treatment. Moreover, we testified that changes in ALT values from baseline to week 24 (dALT2) could act as an novel predictors for reaching HBsAg seroconversion in CHB patients receiving combination therapy. Accurate screening for potentially superior populations for NAs plus PEG-IFN therapy, especially patients with baseline HBsAg levels below 100 IU/mL, may be more conducive to the elimination of HBsAg. In addition, the elevation of ALT levels during treatment also has a certain reference value for predicting HBsAg clearance. Although significant progress has been made in that treatment of CHB over the past two decade, exploring more effective treatments in pursuit of higher cure rates is still a current need, and future research should focus on developing individualized treatment regimens.

## Data Availability

The original contributions presented in the study are included in the article/supplementary material, further inquiries can be directed to the corresponding authors.
